# Clinical and Genetic Characteristics of Mexican Patients with Juvenile Presentation of Niemann-Pick Type C Disease

**DOI:** 10.1155/2014/785890

**Published:** 2014-10-02

**Authors:** Raul E. Piña-Aguilar, Aurea Vera-Loaiza, Oscar F. Chacón-Camacho, Juan Carlos Zenteno, Lilia Nuñez-Orozco, Yuritzi Santillán-Hernández

**Affiliations:** ^1^Department of Medical Genetics, Centro Médico Nacional “20 de Noviembre”, ISSSTE, San Lorenzo No. 502E, Colonia del Valle Sur, Del. Benito Juárez, 03100 México, DF, Mexico; ^2^Department of Genetics-Research Unit, Instituto de Oftalmología “Conde de Valenciana”, Chimalpopoca No. 14, Colonia Obrera, Del. Cuauhtémoc, 06800 México, DF, Mexico; ^3^Department of Biochemistry, Facultad de Medicina, Universidad Nacional Autónoma de México, Avenida Universidad 3000, Ciudad Universitaria, Del. Coyoacán, 04510 México, DF, Mexico; ^4^Department of Adult Neurology, Centro Médico Nacional “20 de Noviembre”, ISSSTE, Félix Cuevas No. 540, Colonia del Valle Sur, Del. Benito Juárez, 03229 México, DF, Mexico

## Abstract

Niemann-Pick type C disease (NPC) is a rare lysosomal disease with a protean presentation, ranging from a fatal neonatal course with visceromegaly to an adult presentation with only neurological or psychiatric symptomatology. In this report we describe the genetic and clinical characteristics of 3 Mexican patients from different families with juvenile presentation of NPC. Clinical examination, imaging of central nervous and gastrointestinal system, and EEG were performed. Genetic studies include sequencing and deletion/duplication analysis of *NPC1* and *NPC2* genes. All patients presented with cognitive impairment, ataxia, and supranuclear vertical gaze palsy; one case had gelastic cataplexy. Also they developed epilepsy and cortical atrophy and two patients had thinning of *corpus callosum*. The 3 patients were compound heterozygotes for *NPC1* sequence variants, including 5 missense and 1 nonsense mutations: p.P1007A and p.F1087L in Case 1; p.Q921P and p.G992R in Case 2; and p.R348∗ and p.V1165M in case 3. Mexican patients with juvenile NPC presented with a variable clinical phenotype and compound heterozygosity. This suggests a relative high frequency of mutation carriers as it is reported for European population. Consequently, clinicians should consider NPC as a diagnosis possibility in any adolescent or young adult patient with juvenile dementia and/or ataxia, even in absence of gelastic cataplexy and supranuclear vertical gaze palsy.

## 1. Introduction

Niemann-Pick type C (NPC) disease (OMIM 257220 and OMIM 607625) is a rare lysosomal disorder with neurological involvement occasioned by abnormal lipid accumulation in cells. NPC disease is caused by mutations in two genes:* NPC1* (95%) and* NPC2* (5%); both genes encode proteins which have a function in lipid traffic from lysosome to membrane [1, 2]. Currently, only molecular studies in* NPC1* or* NPC2* genes or filipin test in fibroblasts can establish the definite diagnosis of NPC [2]. These studies are expensive for screening purposes, making the conclusive diagnosis difficult.

NPC disease has a heterogeneous clinical presentation that varies from an infantile neurovisceral type to just an early dementia in the late-onset forms [1]. The age of presentation is highly variable from the prenatal period to adult age [2]. The late-onset forms of NPC are challenging for diagnosis, because several mental and neurological disorders can overlap with NPC symptomatology [1, 2]. However, patients with the late-onset forms are less affected in relation to organomegaly and the cognitive impairment can progress slowly [2], indicating that potential treatments could be more beneficial in this type of patients.

Reports of the type of presentation and clinical characteristics of patients with NPC in Latin-American countries are limited; the published experience is restricted to Brazil [3]. Furthermore, the NPC International Registry, a pharmaceutical sponsored registry of patients in treatment with miglustat [3], does not include patients from other Latin-American countries.

The first report of a Mexican patient with Niemann-Pick type C disease was an ultra-rare pediatric case with pulmonary proteinosis caused by a homozygous small deletion (c.408_409delAA) in* NPC2* gene [4]. Few mutations in* NPC2* have been described, to date only 20, and they correspond to approximately 5% of mutations found in patients with NPC [1, 2]. Also a Mexican male with late infantile presentation with visceral and neurological symptomatology was described but no genetics studies were reported [5].

In this report we present the clinical and genetic characteristics of three unrelated Mexican patients with juvenile presentation of NPC.

## 2. Material and Methods

Skin fibroblasts were used for LDL-cholesterol esterification, filipin staining (in Cases 1 and 2), and sequencing analysis of all coding regions, exon/intron boundaries, and gross deletion/duplication analysis by multiplex ligation-dependent probe amplification (MLPA) of* NPC1* and* NPC2* genes in all patients. These studies were performed at Mayo Clinic, USA, unless otherwise stated.

For Cases 1 and 2, we performed the analysis of mutations found in* NPC1* in the patients and members of their corresponding families and in Case 3 only in the patient. Genomic DNA was extracted from blood leucocytes using conventional methods in a semiautomatic system (Quickgene, Fujifilm). Each exon was amplified using a specific PCR reaction in which the oligonucleotides were designed to cover the full coding sequence and intron-exon boundaries. The oligonucleotides used for exon 8 were 5-AATAGCAGGGCAGGGTTGAA-3 and 5-GTCTTCTTTCTCTGCTCATCTC-3, for exon 18 were 5-TGGCACCCTCTTATTCTCCA-3 and 5-TCAGTGAGACATTTCAGGCC-3, for exon 20 were 5-GGGAAAGATTATCCCTCAC-3 and 5-TTTCCTGCTTCCACCAAAGG-3, for exon 22 were 5-CCAAAGGAGTCTGACCACTTG-3 and 5-TCTAAGACAGCCAATTCCCC-3, and for exon were 23 5-ATTGGTGTGATGGAGGCCTT-3 and 5-GCCTGAAAGCTTGCAATCCT-3. The PCR products were resolved in agarose gels, purified, and prepared for direct sequencing using the BigDye terminator cycle sequencing kit. The products were bidirectionally analyzed in an ABIPrism 3130 Genetic Analyzer. The obtained sequences were compared with reference sequence of the* NPC1* gene (NG_012795.1).

## 3. Case Reports

### 3.1. Case 1

This was a female, 15 years old, born in Baja California, Mexico, from healthy, nonconsanguineous parents. She was healthy at birth and reached normal developmental milestones. She presented with the first symptoms at the age of 6 years, with poor school performance and frequent falls; then gelastic cataplexy (as in Supplementary Video 1, Available online at: http://dx.doi.org/10.1155/2014/785890, it shows an episode of gelastic cataplexy at 12 years) and dysarthria were added. She received an initial diagnosis of attention deficit hyperactivity disorder (ADHD). Progressively she developed vertical supranuclear gaze palsy (as in Supplementary Video 2, showing supranuclear vertical gaze palsy and experiencing a small head drop at 14 years of age), ataxia (as in Supplementary Video 3, demonstrating ataxia at 14 years of age), dysdiadochokinesia and dysmetria, impaired swallowing, and marked cognitive impairment. She was referred to our hospital to the pediatric neurology department at 11 years of age where further studies were performed. Recently she presented with seizure activity in EEG and worsening of ataxia and dysphagia.

**Figure 1:**
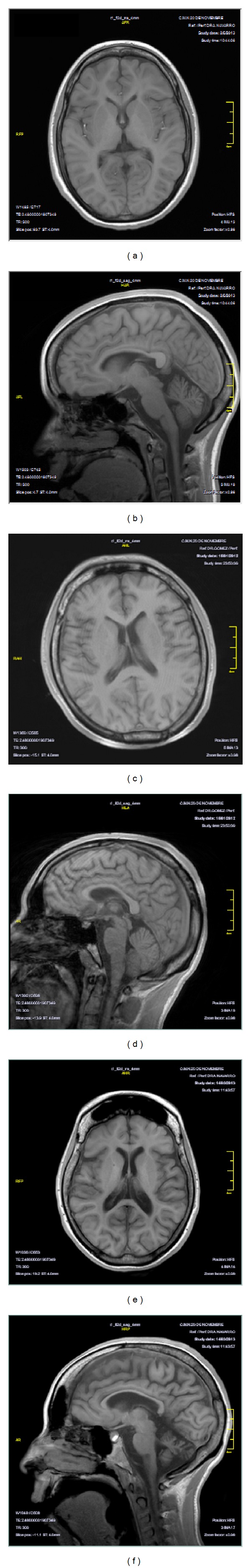
Sagittal and axial T1-weighted MRI images of Case 1 (a, b), Case 2 (c, d), and Case 3 (e, f), showing cortical atrophy (a, c, e) in all and thinning of* corpus callosum* in Case 1 (b) and Case 3 (f), with marked cerebellar atrophy and frontal hemispheres atrophy (e).

Brain MRI at our hospital was initially normal at 11 years of age; later at 14 years of age it showed a slight decrease in volume of both cerebral hemispheres and thinning of corpus callosum, with no abnormalities on cerebellum (Figures 1(a) and 1(b)). EEGs demonstrated voltage asymmetry with low waves and occasionally a generalized paroxysmal activity. When NPC disease was suggested as a possible diagnosis by the genetics department, cholesterol esterification and filipin staining in skin fibroblast were ordered, of LDL-cholesterol esterification (normal >10%) and an abnormal filipin staining. Renal, abdominal, and pelvic ultrasound and sexual steroids were normal.

**Figure 2:**
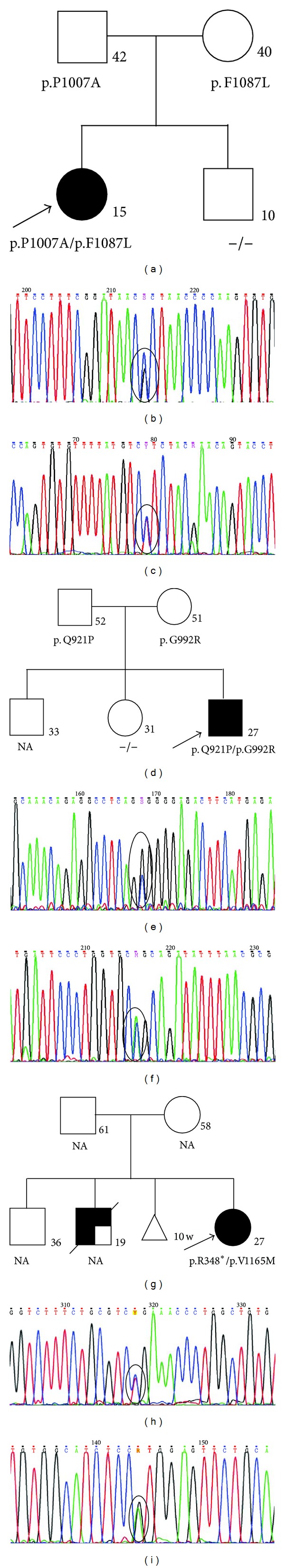
Families' pedigrees of Case 1 (a), Case 2 (d), and Case 3 (g), showing the mutations found. Partial DNA sequence of* NPC1* gene of Case 1 showing the p.P1007A mutation in exon 20 (b) and p.F1087L mutation in exon 22 (c). Partial DNA sequence of* NPC1* gene of Case 2 demonstrating p.Q921P mutation in exon 18 (e) and p.G992R mutation in exon 20 (f). Partial DNA sequence of* NPC1* gene of Case 3 demonstrating p.R348∗ in exon 8 (h) and p.V1165M in exon 23 (i). NA: not available.

Sequencing analysis showed heterozygous missense changes in* NPC1*, one in exon 20 c.3019C>G (p.P1007A) and the other in exon 22, c.3259T>C (p.F1087L). Neither mutations in* NPC2* nor gross deletion/duplications in* NPC1* or* NPC2* were detected. Mutation analysis in the family (pedigree shown in Figure 2(a)) confirmed mutations found in the patient (Figures 2(b) and 2(c)) and demonstrated that the mother is carrier of the c.3019C>G mutation, which is previously reported [6]. The father is carrier of the c.3259T>C mutation, which is a relatively common variant in European population [7] and was recently found in adult presentations of NPC disease [8]. The brother does not carry any of the mutations and is asymptomatic.

Her score in the suspicion index of NPC [9], made retrospectively, was 142 points. Currently, the patient has a score of 14 points in the modified functional disability scale for NPC [10] and she is receiving treatment with miglustat (Zavesca, Actelion Pharmaceuticals) 200 mg every 8 hours.

### 3.2. Case 2

This was a male, 27 years old, born in the State of Mexico, Mexico, from healthy, nonconsanguineous parents. He was healthy at birth and met normal developmental milestones. He presented with symptomatology at the age of 8 years, with poor educational performance, and then at the age of 12 years showed gait abnormalities and facial abnormal movements. Subsequent symptoms included dysarthria, frequent falls, ataxia and dystonia of right limbs (as in Supplementary Video 4, showing ataxia and right limbs spasticity and dystonia at 24 years of age), supranuclear vertical gaze palsy (as in Supplementary video 5, demonstrating supranuclear vertical gaze palsy and slow saccadic movements at 24 years of age), and swallowing difficulties (as in Supplementary Video 6, the patient showing swallowing difficulties with liquids at 24 years). MRI was normal at 22 years of age, but a probable diagnosis of spinocerebellar ataxia was proposed and he was referred to the neurology department at our hospital.

Brain MRI at 24 years of age showed a generalized cortical atrophy, without gross abnormalities on corpus callosum and cerebellum (Figures 1(c) and 1(d)), and EEG showed bilateral frontal paroxysms with generalized slow activity. A muscle biopsy analyzed by light microscopy showed a nonspecific chronic inflammatory process. When evaluated at our genetics department, at the age of 24 years, a metabolic neurodegenerative disease was considered. Abdominal ultrasound demonstrated splenomegaly. At the suspicion of NPC, skin fibroblast culture was performed showing a cholesterol esterification of 0% and abnormal filipin staining.

Nucleotide sequencing found two heterozygous changes in* NPC1*, one in exon 18, c.2762A>C (p.Q921P), and the other in exon 20, c.2974G>C (p.G992R), with no mutations in* NPC2* or gross deletion/duplications in* NPC1* or* NPC2*. We confirmed the reported mutations (Figures 1(e) and 1(f)) in the patient and the mutation analysis of family members (family pedigree in Figure 2(d)) found that the mother is carrier of c.2974G>C missense mutation which is previously reported [11]. This nucleotide position is highly polymorphic with different substitutions reported. Recently this mutation was associated with adult presentations [8]. The c.2762A>C missense mutation was found in the father, which is reported previously [12]. The studied sister does not carry any of the mutations and is asymptomatic.

Suspicion index for NPC [9], performed retrospectively, scored 227 points. The patient initiated miglustat late in progression of NPC disease with a modified functional disability scale score of 17 points [10] by a short period (less than 6 months). He had complicated aspiration pneumonia, which required admission to ICU, pulmonary artificial ventilation, and tracheostomy. He is now relatively stable with tracheostomy and GI tube for feeding and restarted very recently treatment with miglustat 200 mg every 8 hours but shows higher disability score of 21 [10].

### 3.3. Case 3

This was a female, 25 years old, born in Sonora, Mexico, from healthy, nonconsanguineous parents. She had a brother deceased at 19 years of age, who had a clinical progression similar to the patient including symptomatology that began at 10 years of age with neurocognitive impairment, behavioral problems, hearing loss, seizures, and ataxia with diagnosis of schizophrenia. The patient was healthy at birth and had normal developmental milestones, but she was diagnosed with hepatosplenomegaly and leukocytosis at one year of age. Trying to discard acute leukemia, a bone marrow aspiration was performed showing foamy histiocytes; based on this finding a diagnosis of Gaucher disease was established; however she did not have further studies or treatment at that time.

At nine years of age low school performance was noted, requiring special education. She progressively developed behavioral abnormalities including anxiety and aggressive behavior. The patient was diagnosed with schizophrenia and received pharmacological treatment with poor response. She presented with tonic-clonic seizures at 13 years of age, ataxia, frequent falls, dysarthria, and feeding difficulties and progressively developed supranuclear vertical gaze paralysis.

At 15 years of age she was referred to Massachusetts General Hospital, Boston, where a head MRI showed diffused volume loss more prominent at cerebellum while abdominal MRI showed splenomegaly with infarction zones. She had normal lysosomal enzymatic screening panel (for GM1 gangliosidosis, *α*- and *β*-mannosidosis, fucosidosis, Tay-Sachs, Gaucher, metachromatic leukodystrophy, Krabbe, and acid sphingomyelinase). Considering the possibility of NPC disease, a skin biopsy was performed and the analysis by electron microscopy found cytoplasmic polymorphous bodies within nerves and stroma in fibroblasts, which were absent in endothelial, smooth muscle and epidermic cells. Filipin test performed at the Jefferson Medical College was positive. Accordingly, the diagnosis of Niemann-Pick type C was established and the fibroblasts were sent for genetic studies.

Nucleotide sequencing found two heterozygous changes, one in exon 8, c.1042C>T (p.R348∗), and the other in exon 23 c.3493G>A (p.V1165M) of* NPC1*, with neither mutation in NPC2 nor deletions/duplications. The p.R348∗ nonsense mutation was previously reported [13] and its pathogenicity at cellular level on late endosome is well known [14]. The p.V1165M missense mutation was also previously reported in a patient with a classic filipin staining [15]. Both mutations were confirmed in our laboratory (Figures 2(h) and 2(i)); no further mutation analysis was performed in the family (pedigree in Figure 2(g)).

Head MRI at 17 years of age showed diffuse volume loss, most prominently in cerebellum, cortical atrophy and marked atrophy of frontal hemispheres, and an abnormal EEG, with diffuse slowing at the age of 16 years. She developed chronic lithiasic cholecystitis that required gallbladder removal at 22 years of age. At 24 years of age she presented with low platelets and leukopenia treated with splenectomy, which improved after the procedure. Her most recent MRI studies showed cortical atrophy, a thin corpus callosum, and marked cerebellar atrophy (Figures 1(e) and 1(f)).

At 24 years of age, the patient was referred to our hospital to evaluate initiation of therapy with miglustat. Her score in NPC suspicion index [9] performed retrospectively was 227. In our hospital brain MRI confirmed previous findings and EEG found paroxysms with acute-low waves in frontal and temporal brain regions with left predilection. Although progression of the disease is also advanced, with a modified functional disability score of 18 [10], the patient initiated treatment very recently with miglustat 200 mg every 8 hours.

## 4. Discussion

In this report we described the clinical characteristics of Mexican patients with juvenile presentation of Niemann-Pick type C and for the first time mutations in* NPC1* gene in them.

Late-onset presentations of lysosomal disorders can be an arduous diagnosis for the nonmetabolic clinician. The publication of a suspicion index for NPC was designed to help nonmetabolic specialists in diagnosis [9] and promised to be a useful tool. However, it is now clear that not all patients with higher scores have NPC [8]. In a recent multicentric NPC genetic analysis of adult patients with neurological and psychiatric symptoms [8], only 3 patients of 250 (1.2%) had definitive diagnosis of NPC. But several patients in this study had a high score in the NPC suspicion index and clinical characteristics associated with NPC such as supranuclear gaze palsy and splenomegaly confirming that clinical diagnosis of adults is not straightforward.

Juvenile patients with NPC can also be very challenging to diagnose, because they not always present with the classical organomegaly, as in our Case 1. Furthermore, the phenotype can be more related to a cognitive impairment which can be erroneously attributed to developmental delay or to autism/schizophrenia as seen in Cases 1 and 3 and her brother. Interestingly, the 3 patients reported here presented initially with ataxia and cognitive decline with normal neuroimaging studies and only Case 1 had gelastic cataplexy (Supplementary Video 1), which is a feature highly suggestive of NPC diagnosis [2, 3].

The geographical origin of patients and its corresponding genetics differences are relevant for neurogenerative disorders. This is clearly demonstrated for adult-onset disorders like spinocerebellar ataxias, which have a geographical limited distribution, including some with higher incidence and founder effects in Mexico [16, 17]. In the case of lysosomal disorders with late-onset neurological presentation as NPC, little is known about the worldwide prevalence, type of mutations, and frequency of carriers. Furthermore, genetic variability of NPC is very high with more than 340 pathogenic mutations and several polymorphisms reported to date in* NPC1* gene [2].

In this study all patients were compound heterozygotes for* NPC1* mutations, similar to the genotypes reported for the majority of patients affected by NPC from Europe [1]. When, in a given population, the frequency of compound heterozygosity is common, it is probable that the frequency of carriers is very high. No estimates of frequency carriers of* NPC1* or* NPC2* genes are available for the majority of countries [1], but this finding supports the fact that NPC is an underdiagnosed disease. Further studies evaluating allele frequency are urgently needed, especially in Latin populations, to get accurate estimates of NPC incidence and the real burden of this disease.

Although progressive neurological deterioration impacts deeply on the quality of life of the patients and their family, no specific therapy is available and the treatment is mainly supportive. A pharmacological treatment option is a drug named miglustat (Zavesca, Actelion Pharmaceuticals) that works as substrate reduction therapy by inhibiting glucosylceramide synthase. It has been used previously for treatment of neuronopathic Gaucher disease. Miglustat demonstrated in a clinical trial [18] and in open label study [19] that it can stabilize neurological disease in juvenile and adult patients with NPC. It was approved in 2009 by the European Medicine Agency for the treatment of NPC but not by the Food Drug Administration in USA. Other European studies had demonstrated an improvement in dysphagia [20, 21]. Accordingly, the diagnosis and management of NPC vary importantly between Europe and America.

In Mexico, miglustat (Zavesca) was approved in late 2012 and the patients reported here are the first ones in therapy. In the future an early diagnosis of late-onset forms will be required to maximize the benefits of treatment with miglustat in Mexican patients.

An increased awareness of late-onset forms of NPC is needed to get a deeper knowledge of the natural history of disease and to evaluate the clinical efficacy of substrate reduction therapy and of other new therapies on development. Consequently, neurologists and mental health specialists should be sensitive to the characteristics of late-onset presentations of NPC for referral to metabolic/genetic specialists.

## Supplementary Material

Supplementary Video 1: Case 1 with 12 years, the patient was inpatient at our hospital and during a talk she presented the gelastic cataplexy.
Supplementary Video 2: Case 1 with 14 years, showing the supranuclear vertical gaze palsy, with impossibility of pursuit downward movement and experiencing a small dropping of the head.
Supplementary Video 3: Case 1 with 14 years, demonstrating ataxia at the beginning of therapy with miglustat.
Supplementary Video 4: Case 2 with 24 years, showing ataxia and right limbs spasticity and dystonia.
Supplementary Video 5: Case 2 with 24 years, showing supranuclear vertical gaze palsy and slow saccadic movements.
Supplementary Video 6: Case 2 with 24 years, showing swallowing difficulties with liquids.

## References

[1] Vanier MT (2010). Niemann-Pick disease type C. *Orphanet Journal of Rare Diseases*.

[2] Patterson MC, Hendriksz CJ, Walterfang M, Sedel F, Vanier MT, Wijburg F (2012). Recommendations for the diagnosis and management of Niemann-Pick disease type C: an update. *Molecular Genetics and Metabolism*.

[3] Patterson MC, Mengel E, Wijburg FA (2013). Disease and patient characteristics in NP-C patients: findings from an international disease registry. *Orphanet Journal of Rare Diseases*.

[4] Griese M, Brasch F, Aldana VR (2010). Respiratory disease in Niemann-Pick type C2 is caused by pulmonary alveolar proteinosis. *Clinical Genetics*.

[5] Jean-Tron G, Ortega-Ponce F, Islas-Garcia D (2012). Enfermedad de Niemann—Pick tipo C. *Revista Mexicana de Neurociencia*.

[6] Schicks J, vom Hagen JM, Bauer P (2013). Niemann-pick type C is frequent in adult ataxia with cognitive decline and vertical gaze palsy. *Neurology*.

[7] Park WD, O’Brien JF, Lundquist PA (2003). Identification of 58 novel mutations in niemann-Pick disease type C: correlation with biochemical phenotype and importance of PTC1-like domains in NPC1. *Human Mutation*.

[8] Bauer P, Balding DJ, Klünemann HH (2013). Genetic screening for Niemann-Pick disease type C in adults with neurological and psychiatric symptoms: findings from the ZOOM study. *Human Molecular Genetics*.

[9] Wijburg FA, Sedel F, Pineda M (2012). Development of a suspicion index to aid diagnosis of Niemann -Pick disease type C. *Neurology*.

[10] Pineda M, Perez-Poyato MS, O’Callaghan M (2010). Clinical experience with miglustat therapy in pediatric patients with Niemann-Pick disease type C: a case series. *Molecular Genetics and Metabolism*.

[11] Millat G, Marçais C, Tomasetto C (2001). Niemann-Pick C1 disease: correlations between NPC1 mutations, levels of NPC1 protein, and phenotypes emphasize the functional significance of the putative sterol-sensing domain and of the cysteine-rich luminal loop. *The American Journal of Human Genetics*.

[12] Fancello T, Dardis A, Rosano C (2009). Molecular analysis of NPC1 and NPC2 gene in 34 Niemann-Pick C Italian patients: identification and structural modeling of novel mutations. *Neurogenetics*.

[13] Sztolsztener ME, Strzelecka-Kiliszek A, Pikula S, Tylki-Szymanska A, Bandorowicz-Pikula J (2010). Cholesterol as a factor regulating intracellular localization of annexin A6 in Niemann-Pick type C human skin fibroblasts. *Archives of Biochemistry and Biophysics*.

[14] Sztolsztener ME, Dobrzyn A, Pikula S, Tylki-Szymanska A, Bandorowicz-Pikula J (2012). Impaired dynamics of the late endosome/lysosome compartment in human Niemann-Pick type C skin fibroblasts carrying mutation in NPC1 gene. *Molecular BioSystems*.

[15] Sun X, Marks DL, Park WD (2001). Niemann-Pick C variant detection by altered sphingolipid trafficking and correlation with mutations within a specific domain of NPC1. *The American Journal of Human Genetics*.

[16] Magaña JJ, Tapia-Guerrero YS, Velázquez-Pérez L (2014). Analysis of CAG repeats in five SCA loci in Mexican population: epidemiological evidence of a SCA7 founder effect. *Clinical Genetics*.

[17] García-Velázquez LE, Canizales-Quinteros S, Romero-Hidalgo S (2014). Founder effect and ancestral origin of the spinocerebellar ataxia type 7 (SCA7) mutation in Mexican families. *Neurogenetics*.

[18] Patterson MC, Vecchio D, Prady H, Abel L, Wraith JE (2007). Miglustat for treatment of Niemann-Pick C disease: a randomised controlled study. *Lancet Neurology*.

[19] Pineda M, Wraith JE, Mengel E (2009). Miglustat in patients with Niemann-Pick disease Type C (NP-C): a multicenter observational retrospective cohort study. *Molecular Genetics and Metabolism*.

[20] Fecarotta S, Amitrano M, Romano A (2011). The videofluoroscopic swallowing study shows a sustained improvement of dysphagia in children with Niemann-Pick disease type C after therapy with miglustat. *American Journal of Medical Genetics Part A*.

[21] Ginocchio VM, D'Amico A, Bertini E (2013). Efficacy of miglustat in Niemann-Pick C disease: a single centre experience. *Molecular Genetics and Metabolism*.

